# Increasing incidence of macular edema in excessive morning blood pressure surge in patients with retinal vein occlusion

**DOI:** 10.1038/s41598-020-61386-4

**Published:** 2020-03-10

**Authors:** Hyun-Jin Kim, Yong Un Shin, Yonggu Lee, Min Ho Kang, Mincheol Seong, Heeyoon Cho, Ran Heo, Jin-kyu Park, Young-Hyo Lim, Jeong-Hun Shin

**Affiliations:** 1 Division of Cardiology, Department of Internal Medicine, Hanyang University Guri Hospital, Hanyang University College of Medicine, Guri, Republic of Korea; 2 Department of Ophthalmology, Hanyang University Guri Hospital, Hanyang University College of Medicine, Guri, Republic of Korea; 3 Division of Cardiology, Department of Internal Medicine, Hanyang University College of Medicine, Seoul, Republic of Korea

**Keywords:** Cardiology, Medical research, Risk factors

## Abstract

Morning blood pressure surge (MBPS) had been known to be associated with hypertensive target organ injury and vascular events. Retinal vein occlusion (RVO) is also known to be related with underlying cardiovascular risk factors. This study investigated the effect of MBPS on patients with RVO. In total, 76 patients with RVO who had undergone systemic cardiovascular examination including a 24-hour ambulatory blood pressure monitoring, carotid artery intima media thickness, and pulse wave velocity were evaluated between January 2015 and February 2019. The MBPS was calculated as follows: mean systolic blood pressure measured over two hours after awakening minus mean systolic blood pressure measured during the one hour that included the lowest sleep blood pressure. Macular edema was significantly more prevalent in the MBPS group compared with the non-MBPS group. After adjusting for confounding factors, multivariate regression analyses revealed that MBPS independently predicted macular edema in patients with RVO [Odds ratio 4.75, 95% confidence interval 1.136–16.6, p = 0.015]. In conclusion, evaluating blood pressure patterns, especially MBPS, using 24-hour ambulatory blood pressure monitoring may be useful for assessing and predicting ophthalmologic outcome and may facilitate better blood pressure control in patients with RVO.

## Introduction

Retinal vein occlusion (RVO) is a common retinal vascular disease that leads to vision loss^1^. RVO includes branch retinal vein occlusion (BRVO), which is caused by venous thrombosis at an arteriovenous crossing where an artery and vein share a vascular sheath or at the optic disc, and central retinal vein occlusion (CRVO), which results from thrombosis of the central retinal vein and a disturbance in the venous outflow, at the lamina cribrosa or posterior to it^1–3^. The main complication of RVO is macular edema, which can lead visual impairment^4^. Macular edema is the swelling or thickening of the macula due to leakage of fluid and blood components from blood vessels and is a major cause of RVO-associated blindness, angiogenesis, and retinal detachment. Therefore, it is associated with poor ophthalmologic outcomes^5^.

It has been demonstrated that many systemic cardiovascular risk factors such as hypertension, diabetes, dyslipidemia, and systemic inflammatory diseases are associated with RVO; hypertension is one of the most significant risk factors among them^5–7^. In hypertensive patients, nocturnal blood pressure (BP) rise and morning BP surge (MBPS) have been known to be associated with an increased risk of target-organ damage and cardiovascular events^8–12^. Interestingly, RVO has been shown to have circadian variation as well, with event onset time peaking in the early morning hours in previous studies^13,14^. In this study, we evaluated the effect of morning blood pressure surge on patients with RVO.

## Results

### Baseline characteristics

Among 123 patients who had been diagnosed with RVO, 76 patients who were eligible and underwent 24-hour ambulatory blood pressure monitoring (ABPM) were finally included in the analysis. The mean age was 59.3 ± 10.8 years, and 39 (51.3%) patients were males whereas 37 (48.7%) were females. On 24-hour ABPM, average 24-hour systolic blood pressure (SBP) / diastolic blood pressure (DBP) was 133.4 ± 16.9 mmHg/81.7 ± 11.8 mmHg. Average sleep-trough MBPS was 27.0 ± 14.2 mmHg, and average prewaking MBPS was 6.7 ± 19.3 mmHg. The best cut-off points for predicting macular edema, determined through the receiver operating characteristics (ROC) curve analyses, were 29.3 mmHg for sleep-trough MBPS and 14.4 mmHg for prewaking MBPS (Supplementary Fig. S1). Using the cut-off points, both MBPSs were dichotomized to binary variables and the baseline characteristics of patients according to the binary sleep-trough MBPS were presented in Supplementary Table S1.

Baseline characteristics of patients are presented according to the presence of macular edema in Table 1. Patients with macular edema had higher office SBP/DBP, average 24-hour SBP/DBP, average daytime SBP/DBP and average nighttime DBP than those without macular edema. The average nighttime SBP, surge SBP, prewaking SBP, sleep-trough MBPS and prewaking MBPS were not different between the two groups, whereas the binary prewaking MBPS and binary sleep-trough MBPS were more frequent in patients with macular edema. Age, body mass index (BMI), the frequencies of females, comorbidities and antiplatelet agent use, 10-year atherosclerotic cardiovascular disease (ASCVD) risk and the laboratory findings including pulsed-wave velocity (PWV), ankle-brachial indices (ABI), carotid artery intima media thickness (CIMT), and presence of carotid plaques were not significantly different between the two groups.

**Table 1:** Baseline characteristics.

	All	Macular edema (−)	Macular edema (+)	p
	(n = 76)	(n = 27)	(n = 49)	
Age, year	59.3 ± 10.8	60.1 ± 10.3	58.9 ± 11.1	0.621
Female, no. (%)	37 (48.7)	15 (55.6)	22 (44.9)	0.516
Office SBP, mmHg	141.2 ± 20.3	133.1 ± 17.7	145.7 ± 20.4	0.009
Office DBP, mmHg	82.9 ± 14.8	77.5 ± 11.2	85.9 ± 15.8	0.016
BMI, kg/m^2^	25.6 ± 3.2	24.8 ± 3.4	26.0 ± 3.0	0.095
Current smoking, no. (%)	24 (31.6)	8 (29.6)	16 (32.7)	0.989
Comorbidity, no. (%)				
Hypertension	33 (43.4)	12 (44.4)	21 (42.9)	1.000
Diabetes	15 (19.7)	6 (22.2)	9 (18.4)	0.918
Dyslipidemia	18 (23.7)	10 (37.0)	8 (16.3)	0.08
CAD	3 (3.9)	1 (3.7)	2 (4.1)	1.000
CVA	3 (3.9)	3 (11.1)	0 (0.0)	0.078
Medication, no. (%)				
Antiplatelet agents	15 (19.7)	8 (29.6)	7 (14.3)	0.191
10-year ASCVD risk, %	12.9 ± 12.1	12.9 ± 13.5	12.9 ± 11.4	0.984
Ambulatory BP, mmHg				
Average 24-h SBP	133.4 ± 16.9	127.7 ± 16.1	136.5 ± 16.7	0.028
Average 24-h DBP	81.7 ± 11.8	77.8 ± 10.4	83.9 ± 12.1	0.029
Average daytime SBP	135.9 ± 17.3	129.6 ± 15.9	139.4 ± 17.2	0.018
Average daytime DBP	83.3 ± 12.0	79.3 ± 11.0	85.5 ± 12.1	0.031
Average nighttime SBP	125.6 ± 17.9	121.4 ± 18.7	127.9 ± 17.1	0.126
Average nighttime DBP	76.6 ± 12.2	72.7 ± 10.8	78.7 ± 12.6	0.042
Average lowest nocturnal SBP	107.0 ± 16.6	104.9 ± 17.9	108.2 ± 16.0	0.401
Prewaking SBP	126.1 ± 23.0	124.2 ± 18.7	127.1 ± 25.1	0.594
Surge SBP	134.1 ± 17.9	129.1 ± 18.7	136.8 ± 17.1	0.072
Sleep-trough morning surge	27.0 ± 14.2	24.2 ± 10.2	28.6 ± 15.8	0.203
Prewaking morning surge	8.0 ± 19.3	4.9 ± 12.8	9.7 ± 22.1	0.308
Binary sleep-trough MBPS* (%)	29 (38.2)	4 (14.8)	25 (51.0)	0.004
Binary prewaking MBPS^†^ (%)	26 (34.2)	4 (14.8)	22 (44.9)	0.017
Laboratory finding				
Hemoglobin, g/dl	14.2 ± 1.4	13.8 ± 1.3	14.4 ± 1.4	0.069
Cholesterol, mg/dl	197.8 ± 41.6	187.8 ± 35.8	203.3 ± 43.8	0.120
Triglyceride, mg/dl	153.2 ± 79.0	131.5 ± 61.3	165.1 ± 85.5	0.075
LDL-C, mg/dl	118.4 ± 33.8	112.6 ± 38.9	121.5 ± 30.7	0.277
HDL-C, mg/dl	55.1 ± 18.8	54.5 ± 16.8	55.5 ± 20.1	0.829
HbA1C, %	5.9 ± 0.9	5.9 ± 0.8	5.9 ± 1.0	0.978
D-dimer, ng/ml	99.6 ± 70.9	112.0 ± 72.8	93.3 ± 70.2	0.367
Lipoprotein A, mg/dl	16.2 ± 15.4	21.2 ± 20.8	13.5 ± 10.9	0.071
Homocystein, μmol/l	10.2 ± 3.0	10.5 ± 3.0	10.1 ± 3.0	0.612
ABI, average	1.1 ± 0.1	1.1 ± 0.1	1.1 ± 0.1	0.668
PWV, average, mm/s	1684 ± 326	1614 ± 338	1722 ± 317	0.169
Carotid IMT, average, mm	0.7 ± 0.1	0.7 ± 0.1	0.7 ± 0.1	0.371
Carotid plaque, no. (%)	39 (51.3)	12 (44.4)	27 (55.1)	0.516

Data are presented as n (%) or mean (standard deviation).

Abbreviations: ABI, ankle-brachial index; ASCVD, atherosclerotic cardiovascular disease; BMI, body mass index; BP, blood pressure; CAD, coronary artery disease; CVA, cerebrovascular attack; DBP, diastolic blood pressure; HbA1C, hemoglobin A1C; HDL-C, high-density-lipoprotein cholesterol; IMT, intima media thickness; LDL-C, low-density-lipoprotein cholesterol; MBPS, morning blood pressure surge; PWV, pulse wave velocity; SBP, systolic blood pressure.

*Cut-off point ≥29.3 mmHg; ^†^Cut-off point ≥14.4 mmHg.

### Ophthalmological findings including macular edema

Out of the included RVO patients, 54 (71.1%) had branch retinal vein occlusion (BRVO) and 20 (26.3%) had central retinal vein occlusion (CRVO). Macular edema was significantly more common in the MBPS group than in the non-MBPS group (86.2% vs. 51.1%, p = 0.002) (Table 2). When classified according to the types of RVO, macular edema was more common in patients with BRVO (90.5% vs. 51.5%, p = 0.003) in the MBPS group than in the non-MBPS group. However, there was no significant difference in the prevalence of macular edema in patients with CRVO (Supplementary Table S2).

**Table 2:** Ophthalmological findings according to morning surge.

	All	non-MBPS group	MBPS group	p
	(n = 76)	(n = 47)	(n = 29)	
CRVO, no. (%)	20 (26.3)	12 (25.5)	8 (27.6)	0.843
BRVO, no. (%)	54 (71.1)	33 (70.2)	21 (72.4)	0.837
Macular edema, no. (%)	49 (64.5)	24 (51.1)	25 (86.2)	0.002
Baseline CMT, mean (SD), μm	446.2 (186.7)	437.9 (193.8)	458.4 (178.6)	0.651

Abbreviations: BRVO, branch retinal vein occlusion; CRVO, central retinal vein occlusion; CMT, central macular thickness; MBPS, morning blood pressure surge; SD, standard deviation.

### MBPS as a predictor of macular edema in retinal vein occlusion

Univariate logistic regression analysis showed that MBPS was associated with macular edema (the binary sleep-trough MBPS: odds ratio [OR] 5.99, 95% confidence interval [CI] 1.80–19.90, p = 0.004; the binary prewaking MBPS: OR 4.49, 95% CI 1.41–15.60, p = 0.012). Average daytime SBP and average 24-hour SBP were also associated with macular edema in patients with RVO (Table 3). Interestingly, history of diagnosis of and medication for dyslipidemia was negatively associated with macular edema in patients with RVO. We included either the binary sleep-trough MPBS or the binary prewaking MBPS and one of average 24-hour, daytime, and nighttime SBP with other covariates in multivariate logistic regression models, because the binary prewaking MBPS and average daytime and nighttime SBP showed high multicollinearities with the binary sleep-trough MBPS and average 24-hour SBP, respectively. In the multivariate logistic models, either sleep-trough MPBS or prewaking MBPS was repeatedly associated with macular edema, independent of the average SBP and other confounders of atherosclerosis (Table 4). The average 24-hour, daytime and nighttime SBP were all associated with macular edema in the models that included the binary prewaking MBPS, whereas only the average 24-hour SBP among the 3 average SBPs was associated with macular edema in the models that included the binary sleep-trough MBPS.

**Table 3:** Univariate association of various factors with macular edema in patients with RVO.

	OR	95% CI	*p*-value
Binary sleep-trough MBPS*	5.99	1.80–19.9	0.004
Binary prewaking MBPS^†^	4.49	1.41–15.6	0.012
Average 24-hour SBP (per 10 mmHg)	1.41	1.03–1.92	0.032
Average daytime SBP (per 10 mmHg)	1.36	1.01–1.83	0.045
Average nighttime SBP (per 10 mmHg)	1.18	0.87–1.58	0.287
Age (per 5 years)	0.95	0.77–1.19	0.661
Male	1.53	0.60–3.95	0.375
Diabetes	0.79	0.25–2.51	0.687
Hypertension	0.94	0.36–2.42	0.894
Current smoking	1.14	0.12–3.19	0.786
Dyslipidemia	0.33	0.11–0.98	0.047
LDL-C (per 10 mg/dl)	1.08	0.94–1.25	0.275
BMI (Kg/m^2^)	1.15	0.98–1.35	0.093
Average PWV (cm/s)	1.12	0.95–1.30	0.171
Carotid IMT (per 0.1 mm)	1.21	0.80–1.81	0.367

Abbreviations: BMI, body mass index; CI, confidence interval; IMT, intima media thickness; LDL-C, low-density lipoprotein cholesterol; MBPS, morning blood pressure surge; OR, odds ratio; PWV, pulse wave velocity; RVO, retinal vein occlusion; SBP, systolic blood pressure.

*Cut-off point ≥29.3 mmHg; ^†^Cut-off point ≥14.4 mmHg.

**Table 4:** Multivariate logistic regression for the predictors of macular edema in patients with RVO.

model summary	Model 1		Model 2		Model 3		Model 4		Model 5		Model 6	
	Sleep-trough MBPS + Average 24-hour SBP + Covariates^‡^		Sleep-trough MBPS + Average daytime SBP + Covariates^‡^		Sleep-trough MBPS + Average nighttime SBP + Covariates^‡^		Prewaking MBPS + Average 24-hour SBP + Covariates^‡^		Prewaking MBPS + Average daytime SBP + Covariates^‡^		Prewaking MBPS + Average nighttime SBP + Covariates^‡^	
	C-index 0.745; AIC 88.58		C-index 0.681; AIC 92.40		C-index 0.681; AIC 92.40		C-index 0.746; AIC 89.14		C-index 0.729; AIC 90.56		C-index 0.719; AIC 92.20	
	OR (95% CI)	*p*	OR (95% CI)	*p*	OR (95% CI)	*p*	OR (95% CI)	*p*	OR (95% CI)	*p*	OR (95% CI)	*p*
Binary sleep-trough MBPS*	4.74 (1.35–16.6)	0.015	4.71 (1.34–16.4)	0.015	4.77 (1.37–16.6)	0.014	—	—	—	—	—	—
Binary prewaking MBPS^†^	—	—	—	—	—	—	7.12 (1.65–30.7)	0.009	5.88 (1.40–24.7)	0.015	6.93 (1.59–30.2)	0.010
Average 24-hour SBP	1.43 (0.96–2.14)	0.076	—	—	—	—	1.62 (1.06–2.46)	0.025	—	—	—	—
Average daytime SBP	—	—	—	—	—	—	—	—	1.46 (1.00–2.15)	0.052	—	—
Average nighttime SBP	—	—	—	—	—	—	—	—	—	—	1.45 (0.97–2.16)	0.071

Abbreviations: AIC, Akaike Information Criterion; CI, confidence interval; MBPS, morning blood pressure surge; OR, odds ratio; RVO, retinal vein occlusion; SBP, systolic blood pressure.

The multivariate Cox-proportional hazard model was reduced using a backward variable selection method (cut-off criteria p > 0.10).

*Cut-off point ≥29.3 mmHg; ^†^Cut-off point ≥14.4 mmHg.

^‡^Covariates included male, diabetes, hypertension, current smoking, dyslipidemia, LDL levels, BMI, Average PWV and Carotide IMT; None of the covariates were significantly associated with the occurrence of macular edema in all models after the variable selection process.

## Discussion

The results of this study revealed that there is a greater prevalence of macular edema among patients with higher MBPS than among patients with lower MBPS; it also showed that exaggerated MBPS is a predictor of macular edema in RVO patients, independent of ambulatory BP levels and hypertension-mediated organ damage.

RVO is closely associated with systemic cardiovascular diseases including hypertension, dyslipidemia, diabetes, kidney disease, atherosclerosis, myocardial infarction, and stroke^3^. Because patients with RVO exhibit characteristics of cardiovascular disease, their cardiovascular morbidity and mortality rates are also increased^3,15,16^. Among these cardiovascular co-morbidities, hypertension is most commonly seen in patients with RVO and is a well-known risk factor of RVO^6^. RVO is also considered an end-organ damage in patients with hypertension and may be associated with arterial stiffness or carotid atherosclerosis. Moreover, differences in diurnal variation of BP in patients with hypertension, abnormal dipping pattern of nocturnal BP, or presence of MBPS are known to be associated with cardiovascular events^8,9^. A previous study reported that patients with RVO had more non-dipping patterns of nocturnal BP compared with patients without RVO^17^.

BP shows a diurnal variation, with a decrease during sleep and a surge in the morning^18^. In the early morning hours, an abrupt and steep increase in BP occurs, coincident with arousal and awakening from overnight sleep^19^. Normal MBPS is a physiological phenomenon, but an exaggerated MBPS is a cardiovascular risk and there is a substantial ethnic differences in the degree of MBPS^20^. There are compelling evidences suggesting a significant association between MBPS and various surrogate markers of target organ damage as well as cardiovascular events^9–12,21^. Kuwajima *et al*. reported that in 23 elderly hypertensive patients, the SBP change after awaking in the morning was significantly correlated with the left ventricular mass index and the A/E ratio, which represents diastolic function^22^. Kario K. *et al*. also showed that early-morning systolic BP surge was significantly associated with organ damage including left ventricular mass index, CIMT, and PWV^23^. Previous reports also indicated that MBPS is associated with the risk of stroke, independent of the 24-hour BP level in hypertensive patients^24^. In addition, the recent International Database on Ambulatory Blood Pressure Monitoring in Relation to Cardiovascular Outcome Study clearly confirmed that the risk of MBPS for cardiovascular events is only increased in the top tenth percentile of both sleep-trough surge (≥37 mmHg) and prewaking surge (≥28 mmHg)^25^. There is no consensus on the definition or on the threshold of pathological morning BP surge. We defined it in two ways: sleep-trough MBPS and prewaking MBPS. In the present study, both sleep-trough MBPS and prewaking MBPS were significantly associated with macular edema in RVO patients; among the cardiovascular risk factors and BP parameters known to be associated with RVO, MBPS is the strongest predictor of macular edema development. This association was particularly strong for BRVO patients. Although BP was a powerful risk factor of RVO and subsequent macular edema events, the association between MBPS and macular edema was independent of BP. Thus, the identification of a “high morning surge” group in RVO patients may have some clinical significance even after assessment of target organ damage.

Macular edema is a non-specific manifestation associated with retinal vascular disease in general, and a common final pathway of ocular diseases including RVO^26,27^. Macular edema is a clinically important complication in that it causes visual impairment. Although not clearly understood yet, the mechanism of macular edema is known to be associated with the dysfunction of the blood-retinal barrier, inflammation within the vascular wall, and protein and fluid fluxes^27^. Several inflammatory mediators interact with the vascular wall through complex mechanisms^27,28^, which breaks the blood-retinal barrier and increases vascular permeability^29^, and developed macular edema. Although it is yet to be understood in great detail, MBPS, which is one indicator of BP variability, may be related to the aforementioned complex mechanisms, and may contribute in increasing the risk of developing macular edema in patients with RVO. In this study, arterial stiffness or carotid atherosclerosis was not significantly associated with macular edema; however, there were significant associations between MBPS and macular edema. Even after adjusting for ambulatory BP parameters, MBPS was an independent predictor for macular edema in RVO patients. Therefore, the present data indicate that exaggerated MBPS may contribute to the development of macular edema in RVO patents.

To the best of our knowledge, this is the first study designed to evaluate the association between MBPS and macular edema in RVO patients. The level of the MBPS evaluated by 24-hour ABPM may be an important component in the management and treatment of patients with RVO. Additional studies will be needed to characterize BP variability, especially MBPS, in patients with RVO, and clarify its association with treatment response and ocular prognosis.

Some limitations of this study should be considered. The first limitation of this study was that it was designed as a retrospective observational study and a single-center experience. We may have overlooked the presence of RVO in asymptomatic patients, because only relatively symptomatic patients generally visit clinics and are diagnosed with RVO by ophthalmologists. Considering the ratio of patients with BRVO to patients with CRVO in this study, the development of macular edema was more common than the known prevalence of macular edema in RVO, which is around 15% in BRVO eyes^30^, and frequently present in CRVO eyes^31^. Next, we analyzed a relatively small sample size without a control group that was assessed for systemic cardiovascular risk factors without retinal disease. Generalizing our results to a broader group may be limited because we only recruited patients with RVO who underwent cardiovascular risk evaluation without a control group. Further research on a larger sample of RVO patients is needed to confirm the generalizability of our new findings. Finally, the relationship between MBPS and macular edema would be non-linear, given that MBPS within a certain level is physiologic. Although the maximum Youden’s J point would be a reasonable approach to find the optimal cut-off points, the ROC curves analyses for both the sleep-trough and prewaking MBPS did not have sufficiently high C-indexes to provide a confidence in the cut-off points. Since there were no previous reports proposing the optimal cut-off value for the MBPS, further researches are required to determine it.

In conclusion, morning surge in BP was significantly associated with macular edema in patients with RVO. This study suggests that MBPS could be a new therapeutic target for managing and treating RVO and subsequent macular edema in RVO patients. Therefore, evaluating BP patterns using 24-hour ABPM may be useful in assessing ophthalmologic outcomes and may facilitate the initiation of appropriate treatment. Furthermore, it is necessary to control BP delicately to reduce diurnal variation and to improve the outcome of vascular events.

## Methods

### Study design and patients

**Figure 1:**
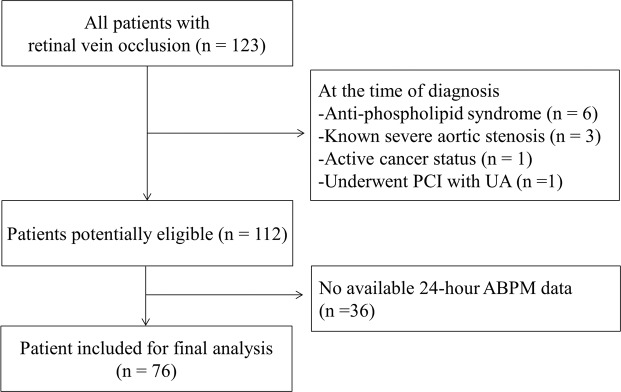
Study population.

Between January 2015 and February 2019, 123 patients who were diagnosed with RVO and received hospital treatment as well as cardiologic consultation were recruited in this study. They underwent 24-hour ABPM and CIMT and PWV measurements. Patients with RVO diagnosed more than 12 months prior to this study were excluded. Among these, six patients who had anti-phospholipid syndrome, three patients who had severe aortic valve stenosis, one patient who had active cancer, and one patient who underwent percutaneous coronary intervention owing to unstable angina were excluded. In addition, 36 patients who had inadequate 24-hour ABPM data were excluded as well (Fig. 1). The study was approved by the Institutional Review Board of Hanyang University and was conducted according to the tenets of the Declaration of Helsinki. Written informed consent was waived by the Institutional Review Board.

### Ophthalmological exams

RVO diagnoses were confirmed by retina specialists. All patients underwent comprehensive ophthalmologic exams such as determination of best-corrected visual acuity, measurement of intraocular pressure and refractive errors, slit-lamp biomicroscopy, and fundoscopy for both eyes. For detailed retinal exams, fundus photography, optical coherence tomography (OCT), and fluorescein angiography were performed. OCT is a non-invasive imaging modality capable of generating *in vivo* cross-sectional retinal and choroidal images. The OCT used in this study was swept-source OCT (DRI OCT Triton, Topcon Corporation, Tokyo, Japan) and we used a 12 × 9 mm 3D wide scan protocol, which comprises 256 B-scans, each comprising 512 A scans (512 × 256 A-scans, 512 A-scans for each of 256 B-scans), and obtained retinal thickness map to identify macular edema. Macular edema was defined when central macular thickness was >300 μm. Patients who had high refractive errors (spherical equivalent >±6), other concomitant ocular diseases (diabetic retinopathy, age-related macular degeneration, uveitis, epiretinal membrane, macular hole in either eye), history of vitreoretinal surgery, and history of ocular trauma were excluded. In addition, patients with a low-quality OCT image that could not be interpreted due to media opacity such as severe cataract were excluded.

### Clinical and laboratory assessments

Patients’ demographic and clinical characteristics were reviewed using electronic records. The following demographic and clinical characteristics were extracted: age, sex, BMI [kg/m^2^], office SBP and DBP, and current smoking status at the time of retinal vascular occlusion diagnosis, as well as traditional cardiovascular risk factors, which included history of diagnosis and treatment of hypertension, diabetes mellitus, dyslipidemia, coronary artery disease, and cerebrovascular attack. Data on antiplatelet drug use were also obtained and 10-year ASCVD risk was calculated from age, history of diabetes, sex, race, smoking status, total cholesterol level, high-density-lipoprotein cholesterol level, SBP, and treatment for hypertension^32^.

The following laboratory data were extracted: hemoglobin, total cholesterol, low-density-lipoprotein cholesterol, hemoglobin A1C, D-dimer, lipoprotein A, and homocysteine, which are known to be associated with thromboembolic events.

### Assessments of ambulatory blood pressure pattern and morning blood pressure surge

ABPM was performed in the non-dominant arm and recorded using an oscillometric device (TM-2430; A&D Company Limited, Tokyo, Japan). ABPM was obtained at 15-minute intervals between 6 a.m. and 10 p.m., and was obtained at 30-minute intervals between 10 p.m. and 6 a.m. Patients were instructed to keep their forearms steady during measurements; they were also instructed to maintain their normal daily activities throughout the duration of the study. Awake time was defined as the actual waking time that patient recorded^33^.Average daytime SBP/DBP was calculated during each patient’s diurnal cycle and average 24-hour and nighttime SBP/DBP were also obtained. The MBPS was calculated in two ways: sleep-trough MBPS, defined as the morning SBP measured over two hours after awakening minus the average of the lowest nocturnal SBP, and prewaking MBPS, defined as the morning SBP measured over two hours after awakening minus the SBP measured over the last two hours before awakening^9,24,25^.

### Assessment of arterial stiffness and subclinical atherosclerosis

For non-invasive measurement of arterial stiffness, brachial-ankle PWV was measured with an oscillometric sphygmomanometric device (VP-1000 plus; Omron Colin, Kyoto, Japan) according to the manufacturer’s recommendation^34^. The procedure was performed with the patient in a supine position after resting for more than five minutes. Both brachia and ankles were wrapped with a cuff. Blood pressure, pulse volume waveform and heart rate were obtained simultaneously. Mean values of the left and right PWVs, ABI were used in the study analysis.

CIMT of both common carotid arteries were measured with a high-resolution ultrasonography iE33 ultrasound system (Phillips Ultrasound, United States) equipped with an 11 L linear transducer. An experienced diagnostic medical sonographer recorded the CIMT measurements using semi-automated edge detection software, which calculates the mean CIMT value from a left and a right common carotid artery at end diastole in a 10-mm segment located 10 mm proximal to the carotid bulb. Mean CIMT values of the left and right carotid arteries were used in the study analysis. Carotid plaque was identified as a focal increase in the CIMT greater than 15 mm or greater than 50% of the surrounding wall^35^.

### Statistical analyses

Given that physiologic levels of MBPS would not increase the risk of macular edema, the sleep-trough MBPS and prewaking MBPS were dichotomized into binary variables using their respective cut-off values, which were derived from the ROC curve analyses. The best cut-off points were determined at the maximum Youden’s J points of their respective ROC curves.

Patients were divided into 2 groups according to the presence of macular edema to compare the baseline characteristics. All categorical data were summarized as frequencies and percentages, and continuous variables were presented as means and standard deviations. The Pearson chi-squared test was used for comparison of categorical variables, and the Fisher’s exact test was used for comparison of categorical variables with 20% or more of the expected cell frequencies ≤5. Student’s *t*-test was used to compare normally distributed continuous variables, and the Mann–Whitney *U*-test was used to compare continuous variables with a skewed distribution.

Univariate logistic regression analyses were used to evaluate the associations of clinical factors and ABPM parameters including 24-hour SBP, daytime SBP, nighttime SBP and MBPS with the presence of macular edema. Multivariate logistic regression analyses were performed to identify variables that were strongly associated with macular edema in the presence of confounding factors. Covariates of the multivariate logistic regression models included sex, past histories of diabetes, hypertension and dyslipidemia, current smoking, serum low density lipoprotein cholesterol levels, BMI, average PWV, carotid IMT, the binary MBPS and the average SBPs in ABPM.

A multivariate logistic regression model including these covariates was reduced to a simpler model through a backward variable selection process (with a cut-off criterion of p > 0.1) to prevent the occurrence of overfitting biases and identify the strong determinants of macular edema. To prevent problems related to multicollinearity, we tested 6 different multivariate logistic regression models, each of which included one of the 2 binary MBPS (sleep-trough and prewaking) and one of the 3 average SBPs in ABPM (24-hour, daytime and nighttime) with the other covariates, and kept the variance inflation factors <2 in the original multivariate model. The goodness of fit of the multivariate logistic regression models were evaluated using the C-index and Akaike information criterion.

All statistical analyses were conducted using the statistical software R-3.5.2 and its packages including “rms”, “ROCR”, “descr” and “tableone”. A p-value < 0.05 was considered significant.

## Supplementary information

Supplementary Information.

## Data Availability

The datasets generated during and/or analyzed during the current study are available from the corresponding author on reasonable request.
