# Supplementary material for: Increasing incidence of macular edema in excessive morning blood pressure surge in patients with retinal vein occlusion
Source: Sci Rep. 2020 Mar 10;10:4420. doi: 10.1038/s41598-020-61386-4 (PMC7064582; doi:10.1038/s41598-020-61386-4)

Supplementary material

Increasing incidence of macular edema in excessive morning blood pressure surge in patients with retinal vein occlusion

Hyun-Jin Kim, MD[1*]; Yong Un Shin, MD[2*]; Yonggu Lee, MD[1,3]; Min Ho Kang, MD[2]; Mincheol Seong, MD[2]; Heeyoon Cho, MD[2†]; Ran Heo, MD[3]; Jin-kyu Park, MD[3]; Young-Hyo Lim, MD[3]; Jeong-Hun Shin, MD[1,3†]

[1] Division of Cardiology, Department of Internal Medicine, Hanyang University Guri Hospital, Hanyang University College of Medicine, Guri, Republic of Korea

[2] Department of Ophthalmology, Hanyang University Guri Hospital, Hanyang University College of Medicine, Guri, Republic of Korea

[3] Division of Cardiology, Department of Internal Medicine, Hanyang University College of Medicine, Seoul, Republic of Korea

[*] These authors contributed equally to this work.

[†] **Co-corresponding authors:**

Jeong-Hun Shin, M.D., Ph.D., Heeyoon Cho, M.D., Ph.D.

Table S1. Baseline characteristics

	All (n =76)	non-MBPS group (n = 47)	MBPS group (n= 29)	*p*-value
Age (year)	59.3±10.8	58.8±10.8	60.2±11.0	0.591
Female	37 (48.7)	23 (48.9)	14 (48.3)	1
Office SBP (mmHg)	141.2±20.3	140.7±20.1	142.1±21.0	0.776
Office DBP (mmHg)	82.9±14.8	82.4±12.6	83.8±18.1	0.701
BMI (kg/m^2)	25.6±3.2	25.6±3.6	25.6±2.3	0.973
Current smoking	24 (31.6)	12 (25.5)	12 (41.4)	0.234
Comorbidity				
Hypertension	33 (43.4)	22 (46.8)	11 (37.9)	0.603
Diabetes	15 (19.7)	10 (21.3)	5 (17.2)	0.894
Dyslipidemia	18 (23.7)	14 (29.8)	4 (13.8)	0.188
CAD	3 (3.9)	1 (2.1)	2 (6.9)	0.667
CVA	3 (3.9)	3 (6.4)	0 (0.0)	0.434
Medication				
Antiplatelet agents	15 (19.7)	9 (19.1)	6 (20.7)	1
10 years ASCVD risk (%)	12.9 (12.1)	11.2±10.1	15.7±14.6	0.115

Ambulatory BP				
Average 24-h SBP (mmHg)	133.4±16.9	131.8±16.5	136.0±17.7	0.298
Average 24-h DBP (mmHg)	81.7±11.8	80.6±11.2	83.6±12.7	0.282
Average daytime SBP (mmHg)	135.9±17.3	133.6±16.5	139.7±18.2	0.133
Average daytime DBP (mmHg)	83.3±12.0	81.7±11.2	85.9±13.1	0.134
Average nighttime SBP (mmHg)	125.6±17.9	125.6±17.7	125.6±18.5	0.999
Average nighttime DBP (mmHg)	76.6±12.2	76.3±12.1	77.0±12.6	0.804
Average lowest nocturnal SBP (mmHg)	107.0±16.6	109.1±17.7	103.7±14.5	0.174
Pre-awakening SBP† (mmHg)	126.1±23.0	125.4±19.8	127.1±27.7	0.757
Surge SBP‡ (mmHg)	134.1±17.9	127.5±16.4	144.7±15.1	<0.001
Sleep-trough MBPS (mmHg)	27.0±14.2	18.4±7.7	41.0±10.7	<0.001
Pre-awakening MBPS (mmHg)	8.0±19.3	2.0±13.6	17.6±23.3	<0.001
Laboratory finding				
Hemoglobin (g/dl)	14.2±1.4	14.2±1.2	14.3±1.6	0.783
Cholesterol (mg/dl)	197.8±41.6	196.4±41.7	200.1±41.9	0.715
Triglyceride (mg/dl)	153.2±79.0	147.7±86.5	162.1±65.5	0.443
LDL-C (mg/dl)	118.4±33.8	115.3±38.1	123.4±25.4	0.312
HDL-C (mg/dl)	55.1±18.8	54.8±15.2	55.8±23.9	0.832
HbA1C (%)	5.9±0.9	5.8±0.8	6.0±1.1	0.408

D-dimer (ng/ml)	99.6±70.9	98.9±66.9	100.6±78.5	0.934
Lipoprotein A (mg/dl)	16.2±15.4	18.1±18.2	13.2±9.0	0.243
Homocystein (µmol/l)	10.2±3.0	10.1±2.3	10.4±3.9	0.731
ABI, average	1.1±0.1	1.1±0.1	1.1±0.1	0.833
PWV, average (mm/s)	1684±326	1641±303	1754±355	0.143
Carotid IMT, average (mm)	0.7±0.1	0.7±0.1	0.7±0.1	0.352
Carotid plaque	39 (51.3)	26 (55.3)	13 (44.8)	0.514

ABI, ankle-brachial index; ASCVD, atherosclerotic cardiovascular disease; BMI, body mass index; BP, blood pressure; CAD, coronary artery disease; CVA, cerebrovascular attack; DBP, diastolic blood pressure; HbA1C, hemoglobin A1C; HDL-C, high-density-lipoprotein cholesterol; IMT, intima media thickness; LDL-C, low-density-lipoprotein cholesterol; MBPS, morning blood pressure surge; PWV, pulse wave velocity; SBP, systolic blood pressure

†SBP during 2-hours before awakening; ‡ SBP during 2-hours after awakening

Table S2. Macular edema in patients with RVO

	All	non-MBPS group	MBPS group	*P*
BRVO	n = 54	n = 33	n = 21	
Macular edema	36 (66.7%)	17 (51.5%)	19 (90.5%)	0.003
CRVO	n = 20	n = 12	n = 8	
Macular edema	13 (65.0%)	7 (58.3%)	6 (75.0%)	0.444

BRVO, branch retinal vein occlusion; CRVO, central retinal vein occlusion; MBPS, morning blood pressure surge

Figure S1. ROC curve analyses

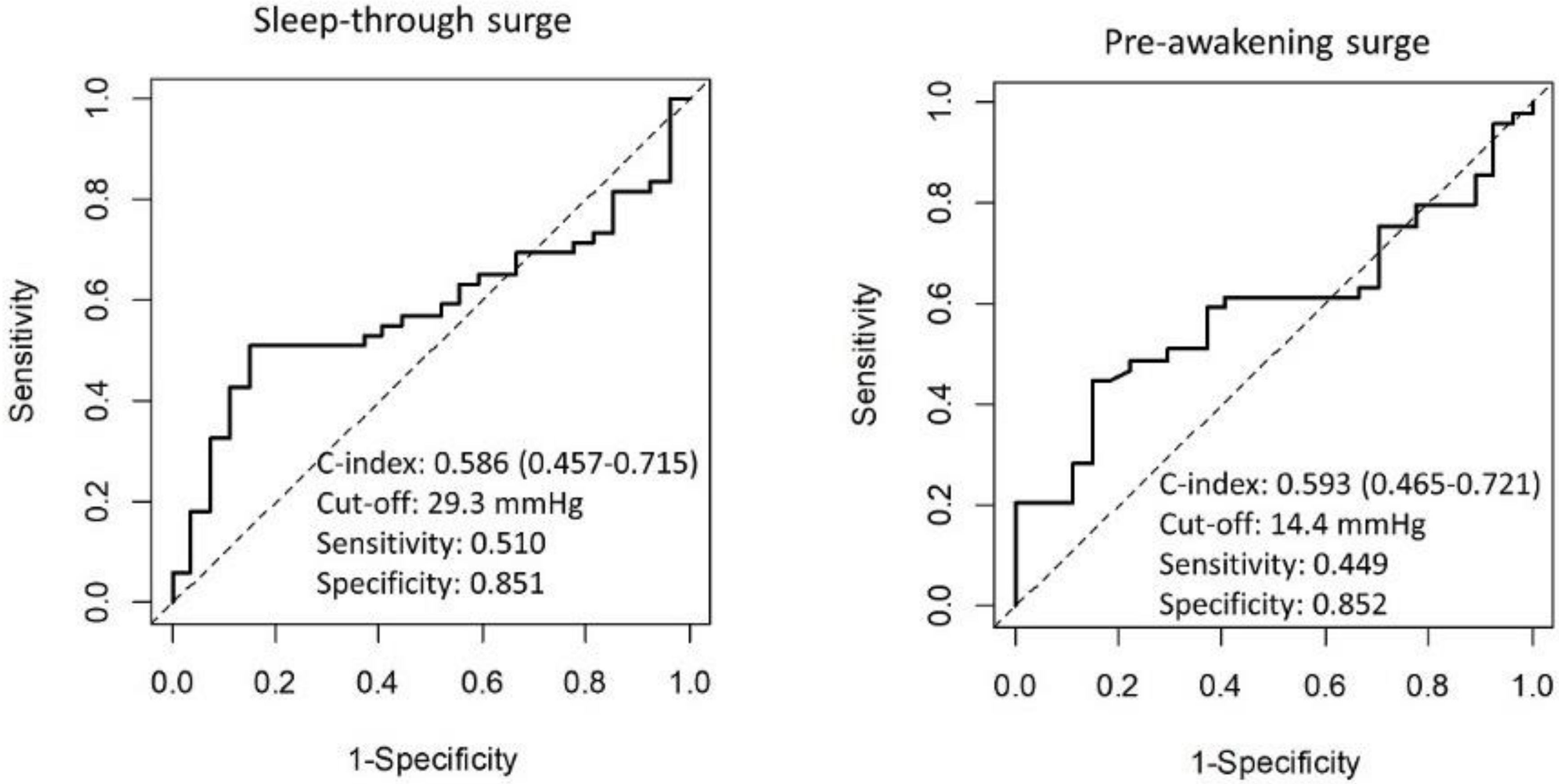

Supplement: Supplementary file 1 — Supplementary Information. [file 41598_2020_61386_MOESM1_ESM.pdf]
